# Identification of Suitable Reference Genes for Gene Expression Normalization in qRT-PCR Analysis in Watermelon

**DOI:** 10.1371/journal.pone.0090612

**Published:** 2014-02-28

**Authors:** Qiusheng Kong, Jingxian Yuan, Lingyun Gao, Shuang Zhao, Wei Jiang, Yuan Huang, Zhilong Bie

**Affiliations:** Key Laboratory of Horticultural Plant Biology, Ministry of Education/College of Horticulture and Forestry, Huazhong Agricultural University, Wuhan, China; Wuhan University, China

## Abstract

Watermelon is one of the major Cucurbitaceae crops and the recent availability of genome sequence greatly facilitates the fundamental researches on it. Quantitative real-time reverse transcriptase PCR (qRT–PCR) is the preferred method for gene expression analyses, and using validated reference genes for normalization is crucial to ensure the accuracy of this method. However, a systematic validation of reference genes has not been conducted on watermelon. In this study, transcripts of 15 candidate reference genes were quantified in watermelon using qRT–PCR, and the stability of these genes was compared using geNorm and NormFinder. geNorm identified *ClTUA* and *ClACT*, *ClEF1α* and *ClACT*, and *ClCAC* and *ClTUA* as the best pairs of reference genes in watermelon organs and tissues under normal growth conditions, abiotic stress, and biotic stress, respectively. NormFinder identified *ClYLS8*, *ClUBCP*, and *ClCAC* as the best single reference genes under the above experimental conditions, respectively. *ClYLS8* and *ClPP2A* were identified as the best reference genes across all samples. Two to nine reference genes were required for more reliable normalization depending on the experimental conditions. The widely used watermelon reference gene *18SrRNA* was less stable than the other reference genes under the experimental conditions. Catalase family genes were identified in watermelon genome, and used to validate the reliability of the identified reference genes. *ClCAT1*and *ClCAT2* were induced and upregulated in the first 24 h, whereas *ClCAT3* was downregulated in the leaves under low temperature stress. However, the expression levels of these genes were significantly overestimated and misinterpreted when *18SrRNA* was used as a reference gene. These results provide a good starting point for reference gene selection in qRT–PCR analyses involving watermelon.

## Introduction

Watermelon (*Citrullus lanatus*), a major Cucurbitaceous crop and the fifth most consumed fresh fruit globally, is an important horticultural crop, and its planting area accounts for 6% of the worldwide area devoted to vegetable production in 2011 (FAOSTAT 2013, http://faostat3.fao.org). Watermelon is the third crop in the Cucurbitaceae family in which the genome has been sequenced, after cucumber and melon. An available genome sequence and large-scale transcriptome data will greatly facilitate molecular biology studies in watermelon [1]–[3].

Gene expression analysis is an effective and widely used approach to elucidate the regulatory networks and identify novel genes in molecular biology. Quantitative real-time reverse transcriptase PCR (qRT–PCR) has become the preferred method for gene expression studies because of its rapidity, sensitivity, and specificity [4]. However, the accuracy of the results obtained by this method depends on accurate transcript normalization using stably expressed reference genes, which allows the regulation of possible non-biological variations when the reference genes are exposed to the same preparation processes as the genes of interest [5], [6]. Therefore, appropriate reference genes should be validated with minimal variability in expression relative to the test samples before qRT–PCR analysis.

However, no validated reference genes have been reported for normalization of gene expression in watermelon as of this writing. *18SrRNA* is frequently used in watermelon as a reference gene for normalization in the fruit [2], [3], root under *Fusarium* wilt infection [7], and leaf under cold stress [8], in qRT-PCR analyses without prior validation. In addition, *actin* was also used in watermelon leaf under water deficit stress [9]. The choice of such traditional genes as references may be inappropriate because their status as housekeeping genes is generally based on methods that are mainly qualitative (e.g., Northern blot) and is inconsistent with the high accuracy associated with qRT-PCR [10]. The disadvantages of using *18SrRNA* as a reference gene include its absence in purified mRNA samples and high abundance compared with target mRNA transcripts, which complicates the accurate subtraction of the baseline value in qRT-PCR data analysis [11].

The use of unstable references can dramatically change the interpretation of an expression pattern of a given target gene, and introduce flaws in the understanding of the function of the gene [12], [13]. Systematic validation of reference genes is essential for producing accurate and reliable data in qRT-PCR analyses, and should be included as an integral component of these analyses [6]. A proper normalization strategy is also among the essential key elements on the Minimum Information for Publication of Quantitative Real-Time PCR Experiments (MIQE) guidelines [14]. The advent of watermelon genome sequence will greatly expedite the completion of studies related to gene expression, whereas the absence of these validated reference genes on watermelon will significantly impede the accurate quantification of gene expression. Validation of suitable reference genes for watermelon can guarantee the accurate quantification of the target genes in qRT-PCR analysis.

In this study, 15 candidate reference genes used in watermelon or validated in other crops were selected, and their transcripts were quantified in the organs and tissues of watermelon under various experimental conditions by qRT-PCR. NormFinder [15] and geNorm [11] were used to identify the suitable reference genes for normalization of gene expression in watermelon.

Catalase family genes are considered as the peroxisomal redox guardians in plants, and the proteins encoded by these genes have relatively specific functions in determining the accumulation of H_2_O_2_, which is an important signal molecule involved in plant development and environmental responses [16], [17]. In *Arabidopsis*, catalase family genes are differentially expressed under different stresses to control reactive oxygen species (ROS) homeostasis [18]. Watermelon is susceptible to many biotic and abiotic stresses during production. However, the functions of catalase family genes in response to environmental stresses on watermelon remain unclear. The reliability of the identified reference genes was further validated by analyzing the expression patterns of catalase family genes under low temperature stress in watermelon leaves using the stable and unstable genes for normalization. The results provide valuable information for suitable reference gene selection in gene expression studies in watermelon.

## Materials and Methods

### Plant Materials and Treatments

The sequenced watermelon inbred line 97103 (*C. lanatus* (Thunb.) Matsum. & Nakai var. *lanatus*) was used as plant material [1]. The seeds were first sterilized with 1.5% sodium hypochlorite, soaked in distilled water for 4 h, and maintained at 30°C for germination. The germinated seedlings were planted in sterilized peat-perlite substrate (2∶1, v/v) and cultured in the greenhouse. Seedlings with two true leaves were used for the following treatments.

The devastating diseases of *Fusarium* wilt and bacterial fruit blotch, which frequently occur on watermelon, were considered as the biotic stresses. Artificial inoculation of bacterial fruit blotch was conducted with 10^8^ cfu⋅mL^–1^ suspension of *pslbtw20* strain (*Acidovorax avenae* subsp. *citrulli*) in accordance with the methods in a previous report [19]. The inoculation of *Fusarium* wilt pathogen was performed as described by Lu et al. [7]. Seedlings were infected with *Fusarium oxysporum* f. sp. *niveum* isolate FON1 by dipping their roots in a conidial suspension of 5×10^6^ spore⋅mL^–1^ for 15 min. The inoculated seedlings were replanted in sterilized substrate and cultured under a 12 h diurnal light cycle at 26°C with 80% to 85% relative humidity inside a controlled environment chamber. Plants were watered every 2 d with 1/2 Hoagland nutrient solution. Root and leaf samples were obtained at 3 d post-inoculation. The remaining seedlings were maintained until typical symptoms of *Fusarium* wilt or bacterial fruit blotch were visible to confirm the success of artificial inoculation.

Low temperature, salinity, and drought, which are the major environmental stresses in watermelon production, were adopted as the abiotic stresses. For low temperature treatment, the seedlings were stored at 10±1°C for 24 h in a controlled environment chamber with a 12 h diurnal light cycle. Seedlings used for salt and drought treatments were transplanted and cultivated hydroponically in 1/2 Hoagland nutrient solution for 5 d in the greenhouse so the seedlings could adapt to the growth environment. For salt treatment, NaCl was gradually added into the nutrition solution until a final concentration of 100 mM was reached, after which the seedlings were cultured for 48 h. For drought treatment, polyethylene glycol 6000 was gradually added into the nutrient solution until a final concentration of 10% was reached, after which the seedlings were cultured for 24 h. Roots and leaves were sampled for each abiotic stress treatment.

Under normal growth conditions, the organs of root, stem, and leaf were collected from the seedlings at the stage of two true leaves. Tendrils were collected from the flowering plants. The day before anthesis, the female flowers were covered by paper bags to prevent natural pollination. On the day of anthesis, flower tissues, including stamen, stigma, petal, and unfertilized ovary, were sampled in the morning. Fruit flesh tissues were collected at 15 and 31 d after pollination.

The biotic and abiotic stress treatments were performed simultaneously, and the seedlings at the stage of two true leaves under normal growth conditions served as controls. Three biological replicates were adopted for the aforementioned treatments, and each replicate contained 15 plants. Samples were randomly collected from five plants for each replicate, immediately frozen in liquid nitrogen, and maintained at −80°C for subsequent RNA extraction.

### Candidate Reference Selection and Primer Design

A total of 15 candidate reference genes were evaluated. These genes were chosen based on their previous use in watermelon or their validation as best reference genes in other crops, including 18S ribosomal RNA (*18SrRNA*), β-actin (*ACT*), clathrin adaptor complex subunit (*CAC*), elongation factor 1-α (*EF1α*), glyceraldehy-3-phosphate-dehydrogenase (*GAPDH*), NADP-isocitrate dehydrogenase (*IDH*), leunig (*LUG*), protein phosphatase 2A regulatory subunit A (*PP2A*), polypyrimidine tract-binding protein 1 (*PTB*), ribosomal protein S (*RPS2*), SAND family protein (*SAND*), α-tubulin (*TUA)*, ubiquitin-conjugating enzyme E2 (*UBC2*), ubiquitin carrier protein (*UBCP*), and yellow-leaf-specific proein8 (*YLS8*).

For each candidate reference gene, blastn was carried out in the Cucurbit Genomics Database (http://www.icugi.org) against watermelon coding DNA sequences (CDS) (v1) using *Arabidopsis* homolog as a query. The CDS of the best hit was retrieved and uploaded to Primer3Plus (http://primer3plus.com/cgi-bin/dev/primer3plus.cgi) for primer design. The product size was set at the range of 80 bp to 150 bp. The forward and reverse primers were intentionally targeted on the adjoining exons, which were separated by an intron. The generated primer pair for each gene was then aligned against all watermelon CDS to confirm its specificity *in silico*. The specificity of the PCR amplification product for each primer pair was further determined by electrophoresis in 2% agarose gel and melting curve analysis. Finally, the watermelon species name abbreviation of ‘*Cl*’ was added as a prefix to the specificity-validated gene to specify the watermelon orthologous gene. For more comparable results, the primer pair of *18SrRNA*, which was previously published, was used in this study [2]. Data on the selected reference genes and their amplification characters are listed in Table 1.

**Table 1:** Information on the selected reference genes.

Gene name	Gene description	Gene ID^a^	Arabidopsis homolog locus^b^	E-value	Forward primer seqence 5′–3′	Reverse primer seqence 5′–3′	Productsize (bp)	Amplificationefficiency(%)	R^2^
*ClACT*	β-Actin	Cla007792	AT3G18780	0	F:CCATGTATGTTGCCATCCAG	R:GGATAGCATGGGGTAGAGCA	140	103.9	0.989
*ClCAC*	Clathrin adaptor complex subunit	Cla020794	AT4G24550	5e-50	F:AATTGTGGTTGATGCTGCAC	R:TGACAGCTGTACCTGGCATC	94	97.2	0.992
*ClEF1α*	Elongation factor 1-α	Cla010539	AT5G60390	0	F: AGCACGCTCTTCTTGCTTTC	R:ACGATTTCGTCGTACCTTGC	115	96.4	0.996
*ClGAPDH*	Glyceraldehy-3-phosphate-dehydrogenase	Cla013454	AT1G16300	2e-80	F:CTGGCAGTACTTTGCCAACA	R:AGGATTGGAGAGGAGGTCGT	87	92.3	0.991
*ClIDH*	NADP-isocitrate dehydrogenase	Cla009135	AT1G65930	2e-71	F:TGGCCTCTTTACCTAAGCACA	R:ATATGCCAGCAGCCTCAAAC	124	94.0	0.991
*ClLUG*	Leunig	Cla022288	AT4G32551	3e-62	F:TTGCTGGTCATTGGATGCTA	R:GCCGAAGCAACTAGACCTGA	138	103.3	0.995
*ClPP2A*	Protein phosphatase 2Aregulatory subunit A	Cla021905	AT1G69960	e-125	F:AAGAGCCCACCAGCTTGTAA	R:TGTTCTCCCCAATCTCAAGG	136	101.0	0.996
*ClPTB*	Polypyrimidine tract-binding protein 1	Cla004906	AT3G01150	2e-15	F:GGAGCAAACAGAAATCAAGC	R:AGCAGGCTCAGAGGAAGATG	133	107.9	0.992
*ClRPS2*	Ribosomal protein S	Cla021565	AT1G04270	3e-67	F:TGGCACTGATCAAGAAGCTG	R:TGATCATATTGCGGAGGTGA	97	98.4	0.996
*ClSAND*	SAND family protein	Cla001870	AT2G28390	4e-42	F:CAATTAGCAGCCGTCAACAA	R:GTTTTGTGAGGGCCAATTTC	100	109.6	0.996
*ClTUA*	α-Tubulin	Cla003129	AT1G64740	e-120	F:CTTGCTGGGAGCTCTATTGC	R:AACGGATTAAAAGCGTCGTG	105	94.6	0.992
*ClUBC2*	Ubiquitin-conjugating enzyme E2	Cla010164	AT2G02760	2e-81	F:CCAAATAAGCCACCGACAGT	R:TCATAGATTGGGCTCCATTG	118	93.7	0.991
*ClUBCP*	Ubiquitin carrier protein	Cla010163	AT1G14400	6e-81	F:ACCAACAGTCCGCTTTGTGT	R:ATTGGGCTCCACTGATTTTG	101	96.6	0.995
*ClYLS8*	Yellow-leaf-specific proein8	Cla020175	AT5G08290	e-78	F:AGAACGGCTTGTGGTCATTC	R:GAGGCCAACACTTCATCCAT	83	94.4	0.995
*Cl18SrRNA* c	18S ribosomal RNA				F:AGCCTGAGAAACGGCTACCACATC	R:ACCAGACTCGAAGAGCCCGGTAT		92.1	0.995

a Watermelon gene ID in Cucurbit Genomics Database (http://www.icugi.org);

b Arabidopsis gene ID in TAIR database (http://www.arabidopsis.org/);

c The primer pair of *18SrRNA* previously published was used here (Guo et al. 2011).

### Total RNA Extraction, cDNA Synthesis, DNA Isolation, and PCR Amplification

The eleven golden rules of qRT–PCR were adopted as guidelines for RNA isolation, cDNA synthesis, and subsequent qRT–PCR analysis [20]. Total RNA was isolated using TransZol (TransGen) according to the manufacturer’s instructions. The integrity of RNA was determined by electrophoresis in 2% agarose gel. The quantity and purity of RNA were determined using a NanoDrop™ 2000 spectrophotometer (Thermo Scientific). Only high-quality samples in which A_260_/A_280_>1.8 and A_260_/A_230_>2.0 were used for subsequent cDNA synthesis. Genomic DNA elimination and cDNA synthesis were conducted using PrimeScript RT Reagent Kit with genomic DNA (gDNA) Eraser (Perfect Real Time, TaKaRa) according to the manual. For each sample, 1 µg of total RNA was used for each 20 µL reverse transcription reaction system. Genomic DNA was isolated from the leaves using Plant Genomic DNA Kit (Tiangen), and PCR amplifications were conducted using 2× PCR Reagent (Tiangen) according to the manual. The amplification products were resolved on 2% agarose gel.

### qRT–PCR Analysis

qRT–PCR was carried out on a LightCycler480 System (Roche) using TransStart Top Green qPCR SuperMix (TransGen). Reactions were performed using a total volume of 10 µL, which contained 100 ng of cDNA template, 0.2 µM each primer, and 1×Top Green qPCR SuperMix. The PCR cycling conditions were as follows: 94°C for 30 s, followed by 40 cycles of 95°C for 5 s, 55°C for 15 s, and 72°C for 10 s. The melting curve was recorded after 40 cycles to verify primer specificity by heating from 65°C to 95°C. Two technical replicates were adopted for each sample. Controls that were obtained without a template were included. For each gene, the full sample set in a replication was run on the same plate to exclude any technical variation. Amplification efficiencies for all primer pairs were evaluated using the serial fivefold dilutions of the pooled cDNA (500, 100, 20, 4, and 0.8 ng).

### Data Analysis

Expression levels of the tested reference genes are determined by crossing point (Cp) values. The amplification efficiency (E) for each reference gene was calculated according to the following equation: E (%) = (10^−1/slope^–1)×100, where the slope is the standard curve slope calculated by the LightCycle 480 system. geNorm [11] and NormFinder [15] were used to assess the expression stability. Based on the principle that the expression ratio between two ideal reference genes should be invariable if the genes are stably expressed across the investigated sample set, geNorm calculates the gene expression stability value M, which is the average pairwise variation of a given gene with all other candidate reference genes, for each tested reference gene. The candidate reference genes are then ranked based on M value, and the gene with lower M value is considered to have higher expression stability. The least stable genes with the highest M are excluded stepwise until only the two most stable genes remain. The best two genes are ranked without distinguishing between them [11]. NormFinder calculates the expression stability for each single reference gene using a model-based approach with consideration of variations across groups. Lower stability value means higher expression stability of the gene [15]. For both algorithms, the input data should be on a linear scale. Therefore, the raw Cp values were transformed to relative quantities *Q* using *Q* = 2^(minCp–sampleCp)^ equation. R package (http://www.r-project.org/) was used to draw the plots.

To identify more stable genes under specific experimental conditions, all 20 samples were subdivided into three subsets based on their origins. The organ and tissue subset comprised organs and tissues from root, leaf, stem, tendril, pistil, stamen, petal, unfertilized ovary, and fruit flesh tissues at 15 and 31 d after pollination under normal growth conditions (10 samples); the biotic stress subset comprised roots and leaves collected from plants infected with *Fusarium* wilt and bacterial fruit blotch and the control (six samples); and the abiotic stress subset comprised roots and leaves collected from plants subjected to drought, salt, and low temperature stresses and the control (eight samples).

### Normalization of Catalase Family Genes

To validate the reliability of the identified reference genes, the relative expression levels of watermelon catalase family genes under low temperature stress were analyzed. To identify the catalase family genes in the watermelon genome, the Hidden Markov Model of the catalase family (PF00199) was downloaded from the Pfam database (http://pfam.sanger.ac.uk/) and used as a query against the watermelon proteins (watermelon_v1.pep, ftp://www.icugi.org) using HMMER [21]. The primers were designed using Primique [22], which can design specific PCR primer pairs for each gene in a family. Specificity of the primers was further checked by electrophoresis in agarose gel and melting curve analysis. Low temperature treatment was conducted as mentioned above, and samples from leaves were obtained at 0, 12, 24, 36, and 48 h. Three biological replicates were adopted for each treatment. The transcripts of catalase genes were quantified by qRT–PCR, and two technical replicates were adopted for each sample. The relative expression levels were calculated using the 2^−ΔΔCp^ method. The single, pair of, and multiple best reference genes identified in this study, as well as the widely used reference gene *18SrRNA* in watermelon, were used as reference genes for normalization.

## Results

### Amplification Specificity and Efficiency for Each Candidate Reference Gene

To identify suitable reference genes for watermelon, 15 candidate reference genes were selected, including 14 reference genes previously identified as the most stable genes in other crops and the most frequently used reference gene *18SrRNA* in watermelon in qRT–PCR analyses. The watermelon orthologous genes were obtained by searching for watermelon CDS using *Arabidopsis* genes as queries. The best hit for each query was selected, and the same annotation as that in the *Arabidopsis* query was found in the watermelon genome database for each watermelon orthologous gene. Information on the selected reference genes is listed in Table 1.

To prevent the interference of gDNA contamination and pseudogene on qRT–PCR results, the forward and reverse primers were specifically located on the neighboring exons during primer design. The generated primer pairs were aligned with all watermelon CDS using blastn to confirm their specificity on a genomic scale *in silico*. When the target reference gene was the only output result of blastn, the primer pair was selected. For more comparable results, a primer pair of *18SrRNA* published in previous qRT–PCR analyses on watermelon was used in the present study.

**Figure 1:**
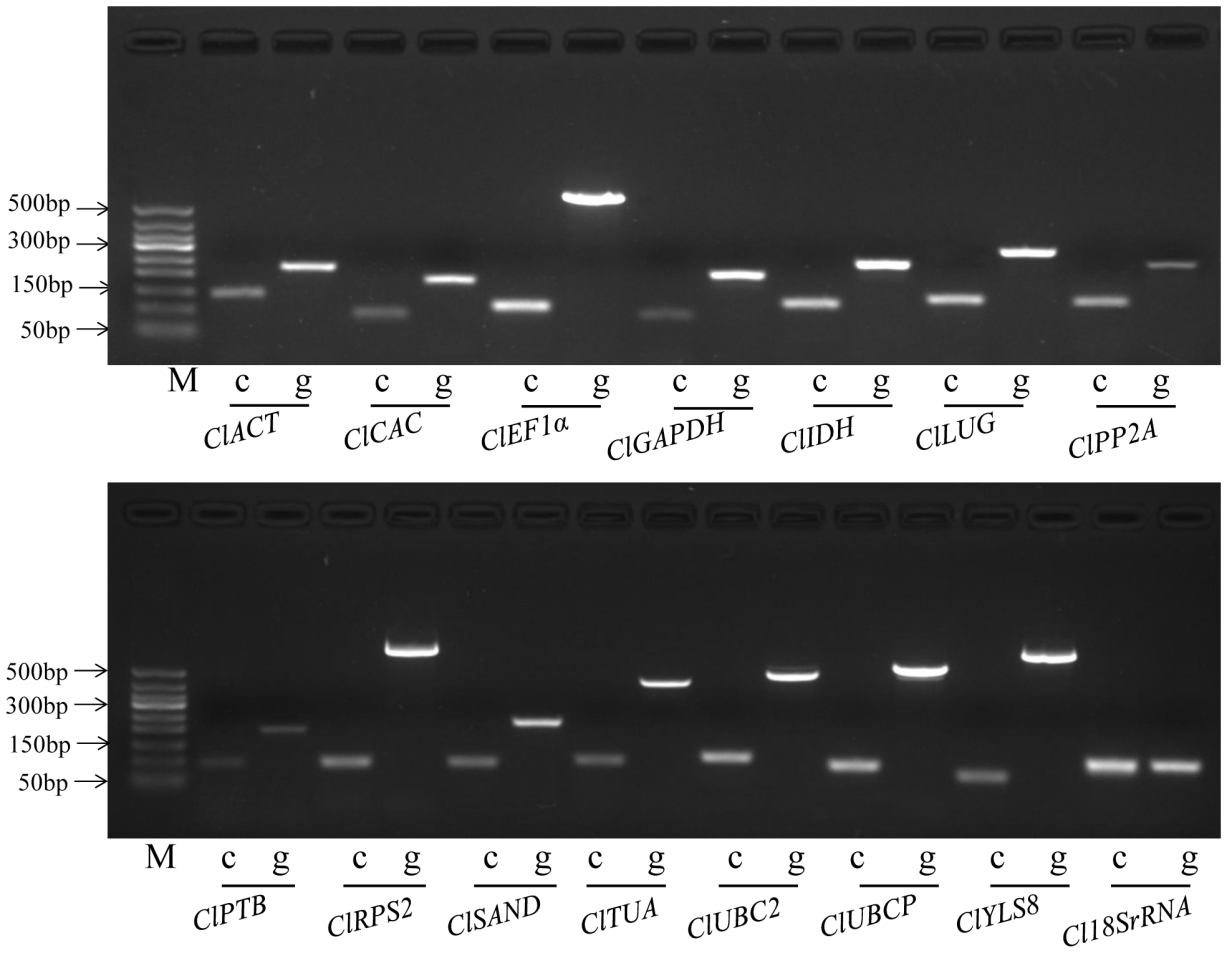
PCR amplification patterns of the candidate reference genes using cDNA and genomic DNA as templates, respectively, as visualized on 2% agarose gel. ‘c’ represents the use of cDNA as template. ‘g’ indicates the use of genomic DNA as template. ‘M’ represents the marker of 50 bp ladder.

PCR amplification specificity for each candidate reference gene was checked by electrophoresis in agarose gel using cDNA and gDNA as templates. As shown in Fig. 1, the primer pair for each reference gene amplified a specific product on both cDNA and gDNA templates. Amplicons with different sizes were observed between the cDNA and gDNA templates for the tested candidate genes, except for *18SrRNA*, which demonstrates the success of primer design and confirms that gDNA contamination did not occur in the cDNA samples. Melting curve analyses were also conducted for all the primer pairs. As shown in Fig. S1, the presence of a single peak with no visible primer-dimer formation further confirmed the specific amplification for each reference gene. Meanwhile, no signals were detected in the no-template controls. The specificity-validated primer sequences and amplification characters for the candidate reference genes are summarized in Table 1.

The qRT–PCR efficiency was determined for each primer pair by standard curve analysis, ranging from 92.1% (*Cl18SrRNA*) to 109.6% (*ClSAND*). The determination coefficients (R^2^) of the standard curve regression equation varied from 0.989 for *ClACT* to 0.996 for *ClEF1α*, *ClPP2A*, *ClRPS2*, and *ClSAND* (Table 1). The aforementioned results prove that specific and high-efficiency qRT–PCR systems were established for the selected reference genes.

### Expression Profiles of the Candidate Reference Genes

**Figure 2:**
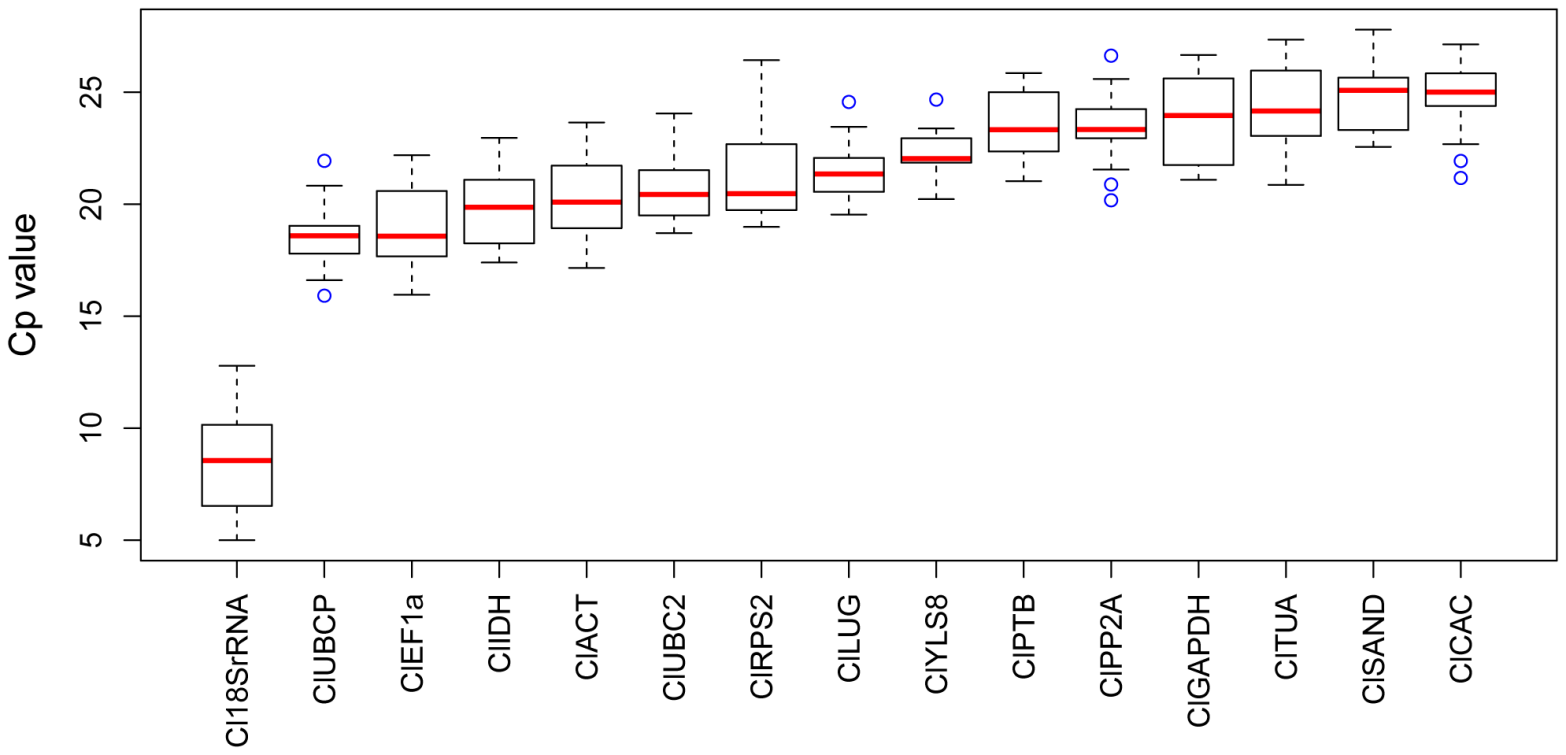
Expression profiles of the tested reference genes in raw Cp values in all 20 samples. Expression data are displayed as Cp values for each reference gene in all samples. The line across the box is the median. The boxes indicate the 25/75 percentiles. Whisker caps indicate the minimum and maximum values. The circles represent the outliers.

Expression levels of the 15 candidate reference genes were measured in the 20 samples collected from watermelon organs and tissues under normal growth conditions and biotic and abiotic stresses by qRT–PCR, and presented as Cp values. Expression variations for these genes across the 20 samples are shown in Fig. 2. Different levels of transcription abundance were observed among these genes. *ClCAC*, which had the highest mean Cp value of 24.8, was expressed at the lowest level among the candidate reference genes. By contrast, *Cl18SrRNA* with the lowest average Cp value of 8.4 exhibited the highest transcription abundance. However, the expression levels for most of the genes were comparable and ranged from 18 to 24 cycles. None of the tested reference genes exhibited a constant expression level among the samples. The variability of Cp values in the 20 samples was highest for *Cl18SrRNA* and *ClRPS2*, whereas *ClYLS8* and *ClPTB* showed the lowest gene expression variations (Fig. 2). Five to six cycles of expression variations were observed for most candidate reference genes. However, given the variations in the amount of starting materials between the samples and subsequent operations of qRT–PCR, a direct comparison of the raw Cp values did not result in an accurate estimate of the expression stability of each reference gene. Therefore, the expression variation must be evaluated by more powerful methods.

### Expression Stability Analyses

**Figure 3:**
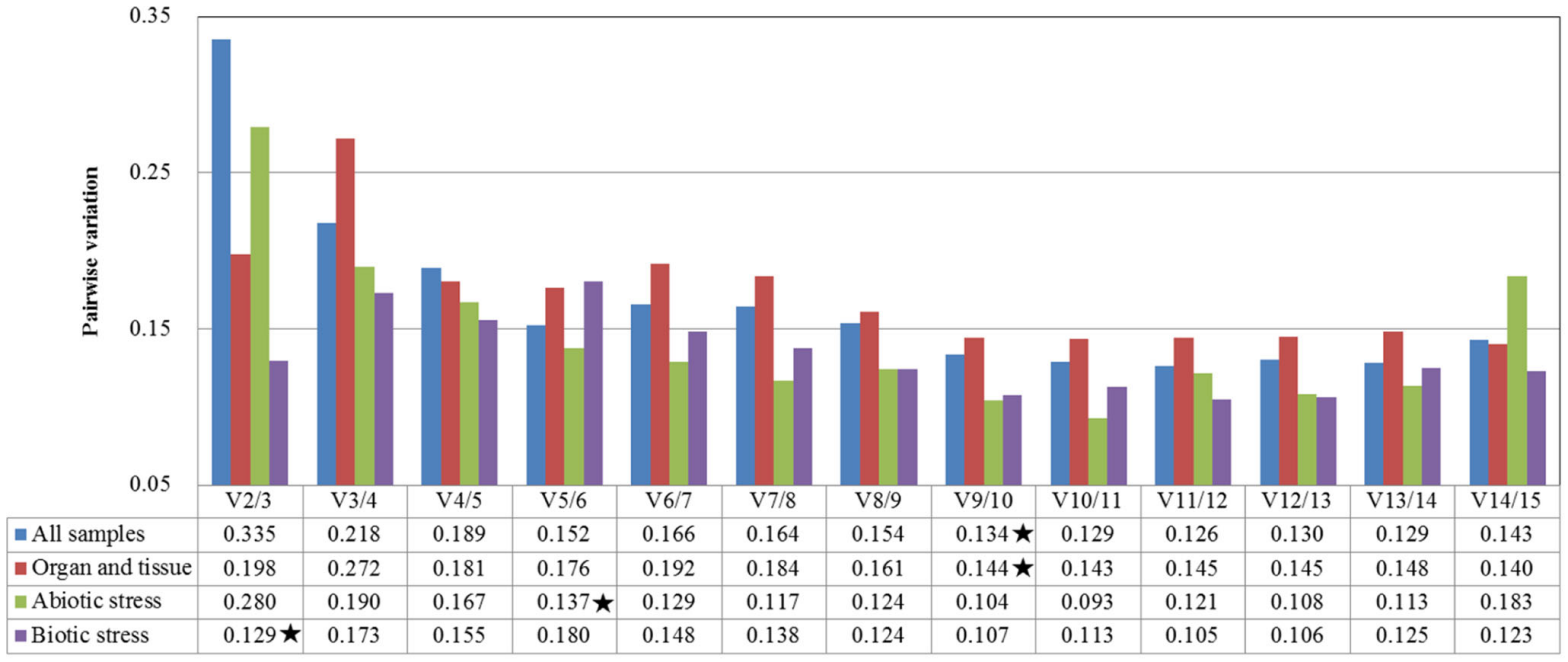
Pairwise variation analyses of candidate reference genes in different sample sets. Pairwise variation (V) was calculated by geNorm to determine the minimum number of reference genes required for accurate normalization in different sample sets. “★” indicates the value of pairwise variation less than the recommended 0.15 for each sample set.

geNorm and NormFinder were used to evaluate the stability of the candidate reference genes. The ranks of the selected reference genes were determined by geNorm and are listed in Table 2. When all 20 samples were considered, *ClYLS8* and *ClPP2A* showed the lowest average expression stability value (M = 0.763), whereas *ClUBC2* showed the highest M value of 1.743. These results suggest that *ClYLS8* and *ClPP2A* had the most stable expression, whereas *ClUBC2* had the highest level of expression variation. Furthermore, pairwise variation between two sequential normalization factors (NFs) containing an increasing number of genes was also calculated using geNorm to determine the optimal number of genes required for normalization. A high pairwise variation indicates that the added gene had a significant effect and should preferably be included in the calculation of a reliable NF. The cut-off value of 0.15 was proposed, below which the inclusion of an additional reference gene is not required [11]. The results are illustrated in Fig. 3. The analysis showed that the pairwise variation V8/9 was higher than 0.15 (V = 0.154), whereas V9/10 was 0.134, which indicates that nine genes were required for more reliable normalization of target genes across the 20 samples. The results of expression stability, as evaluated by NormFinder, are summarized in Table 3. Compared with the geNorm results, *ClYLS8* with the lowest stability value of 0.386 was also ranked as the most stable reference gene by NormFinder, and *ClUBC2* with the highest stability value of 1.467 was identified as the least stable reference gene.

**Table 2:** Watermelon reference genes ranked according to their expression stability as determined by geNorm in different sample sets.

All samples	Stabilityvalue (M)	Organ andtissue	Stability value (M)	Abiotic stress	Stability value (M)	Biotic stress	Stability value (M)
*ClYLS8* *	0.763	*ClTUA* *	0.348	*ClEF1α* *	0.481	*ClCAC* *	0.320
*ClPP2A* *	0.763	*ClACT* *	0.348	*ClACT* *	0.481	*ClTUA* *	0.320
*ClACT* *	0.970	*ClEF1α* *	0.529	*ClUBCP* *	0.742	*ClEF1α*	0.386
*ClEF1α* *	1.000	*ClPP2A* *	0.831	*ClPTB* *	0.809	*ClRPS2*	0.550
*ClTUA* *	1.049	*ClYLS8* *	0.913	*ClIDH* *	0.859	*ClYLS8*	0.665
*ClCAC* *	1.075	*ClCAC* *	1.014	*ClRPS2*	0.895	*ClUBCP*	0.826
*ClUBCP* *	1.152	*ClUBCP* *	1.146	*ClLUG*	0.934	*ClSAND*	0.918
*ClPTB* *	1.239	*ClPTB* *	1.271	*ClTUA*	0.977	*ClPTB*	0.989
*ClLUG* *	1.318	*ClSAND* *	1.362	*ClCAC*	1.042	*ClIDH*	1.044
*ClSAND*	1.375	*ClGAPDH*	1.430	*ClYLS8*	1.083	*ClLUG*	1.079
*ClIDH*	1.430	*ClLUG*	1.503	*ClPP2A*	1.113	*ClUBC2*	1.131
*ClGAPDH*	1.491	*ClIDH*	1.585	*ClSAND*	1.194	*Cl18SrRNA*	1.175
*ClRPS2*	1.565	*Cl18SrRNA*	1.675	*ClGAPDH*	1.259	*ClPP2A*	1.234
*Cl18SrRNA*	1.640	*ClUBC2*	1.775	*Cl18SrRNA*	1.330	*ClACT*	1.328
*ClUBC2*	1.743	*ClRPS2*	1.862	*ClUBC2*	1.536	*ClGAPDH*	1.419

*represents the multiple reference genes determined by pairwise variation analysis which are presented in Fig. 3. The recommended threshold of 0.15 is adopted.

**Table 3:** Watermelon reference genes ranked according to their expression stability as determined by NormFinder in different sample sets.

All samples	Stability value	Organ and tissue	Stability value	Abiotic stress	Stability value	Biotic stress	Stability value
*ClYLS8*	0.386	*ClYLS8*	0.353	*ClUBCP*	0.277	*ClCAC*	0.064
*ClUBCP*	0.492	*ClPP2A*	0.479	*ClRPS2*	0.340	*ClTUA*	0.271
*ClPP2A*	0.572	*ClUBCP*	0.535	*ClTUA*	0.432	*ClRPS2*	0.308
*ClEF1α*	0.652	*ClPTB*	0.713	*ClLUG*	0.473	*ClYLS8*	0.368
*ClCAC*	0.659	*ClACT*	0.759	*ClYLS8*	0.515	*ClEF1α*	0.403
*ClPTB*	0.706	*ClEF1α*	0.766	*ClACT*	0.569	*ClUBC2*	0.649
*ClTUA*	0.731	*ClTUA*	0.829	*ClCAC*	0.574	*ClUBCP*	0.660
*ClACT*	0.770	*ClCAC*	0.834	*ClPTB*	0.650	*ClSAND*	0.684
*ClLUG*	0.874	*ClSAND*	0.951	*ClIDH*	0.664	*Cl18SrRNA*	0.767
*ClSAND*	0.898	*ClLUG*	1.020	*ClPP2A*	0.682	*ClPP2A*	0.802
*ClIDH*	0.967	*ClGAPDH*	1.062	*ClEF1α*	0.692	*ClPTB*	0.849
*ClGAPDH*	1.050	*ClIDH*	1.179	*ClSAND*	0.949	*ClIDH*	0.958
*ClRPS2*	1.103	*Cl18SrRNA*	1.218	*ClGAPDH*	0.970	*ClLUG*	0.999
*Cl18SrRNA*	1.247	*ClUBC2*	1.364	*Cl18SrRNA*	1.196	*ClACT*	1.164
*ClUBC2*	1.467	*ClRPS2*	1.438	*ClUBC2*	1.898	*ClGAPDH*	1.265

To determine more stable reference genes under specific experimental conditions, the 20 samples were further divided into three different subsets, as described in the data analysis section, and the experimental condition-specific reference genes were identified for each subset.

In the organ and tissue subset, which included different vegetative and reproductive organs or tissues of watermelon under normal growth conditions, *ClTUA* and *ClACT* were identified as the best pair of reference genes by geNorm (Table 2), and as many as nine genes were satisfactory for normalization because the pairwise variation value V9/10 was less than 0.15 (Fig. 3). NormFinder identified *ClYLS8* as the best reference gene for this subset (Table 3). Both algorithms identified *ClRPS2* as the gene with unstable expression.

In the abiotic stress subset, *ClEF1α* and *ClACT* were identified as the best pair of reference genes by geNorm, and five genes, namely, *ClEF1α*, *ClACT*, *ClUBCP*, *ClPTB*, and *ClIDH*, comprised the optimal reference genes for more accurate normalization. *ClUBCP* was also identified as the most suitable reference gene by NormFinder. Similarly, *ClUBC2* was identified as the least stable gene by both programs.

In the biotic stress subset, geNorm ranked *ClCAC* and *ClTUA* as the best reference genes, which were sufficient for more reliable normalization because the pairwise variation value V2/3 (0.129) was below the cut-off value of 0.15. *ClCAC* was also ranked as the best reference gene by NormFinder. Both geNorm and NormFinder identified *ClGAPDH* as the least stable reference gene.

*Cl18SrRNA*, the most frequently used reference gene in watermelon in qRT–PCR analyses, ranked from second to seventh from the bottom in all 20 samples and different subsets both by geNorm and NormFinder, which indicates that this gene was unsuitable for normalization in qRT–PCR analyses in watermelon.

### Expression Profiles of Catalase Family Genes

Using the Hidden Markov Model of the catalase family, four genes (*Cla023447*, *Cla023448*, *Cla021932*, and *Cla003205*) were identified in watermelon genome using HMMER, with the threshold E-value <0.01. These genes were further confirmed by searching *Arabidopsis* proteins in the TAIR database (http://www.arabidopsis.org/) using blastp. The genes *Cla023448*, *Cla023447*, and *Cla021932* significantly matched three members of *Arabidopsis* catalase family genes, and were designated as *ClCAT1*, *ClCAT2*, and *ClCAT3*, respectively, according to the best match with their *Arabidopsis* counterparts. However, the best hit for *Cla003205* was *AT5G38120*, with the annotation of AMP-dependent syntheses (E-value = 8e−17). Consequently, *Cla003205* was dropped from subsequent analyses. The primer pair for each watermelon catalase gene was specifically designed to avoid amplifying multiple transcripts in qRT–PCR analysis. Information regarding watermelon catalase family genes and their primers is summarized in Table S1. Amplification specificity of the primers was further confirmed by electrophoresis in agarose gel and melting curve analysis (Fig. S2).

**Figure 4:**
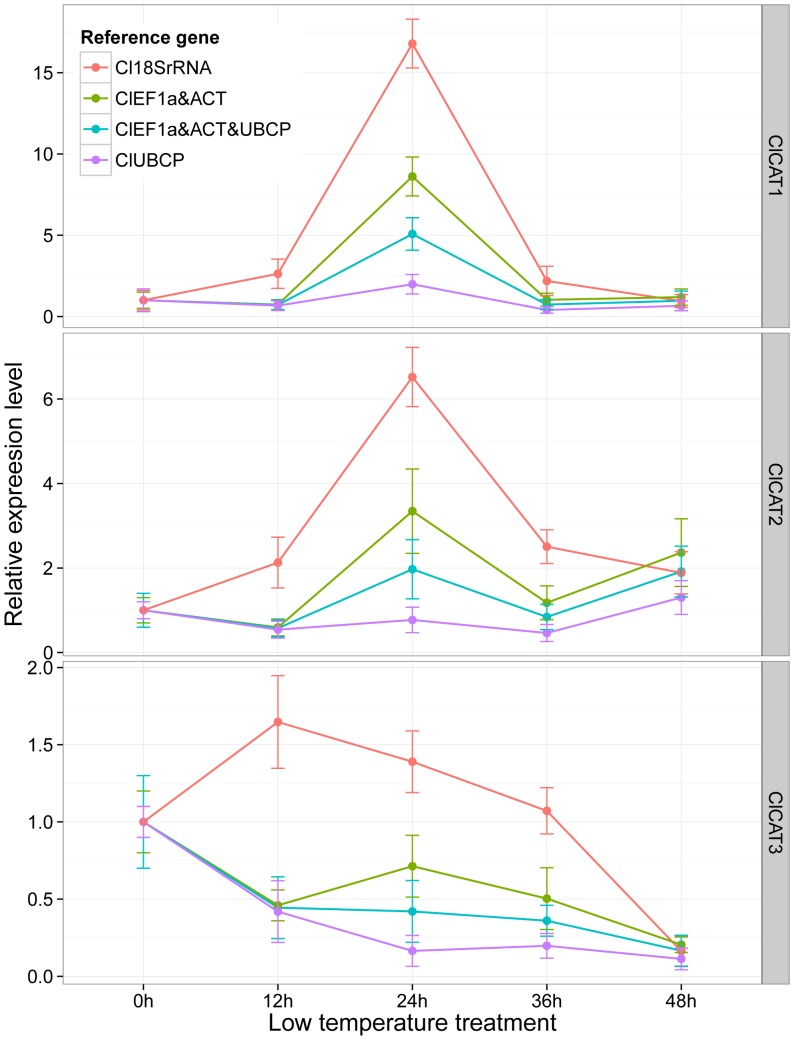
Expression profiles of catalase family genes in watermelon leaves under low temperature stress. Geometric mean was calculated for the pair of best reference genes and multiple reference gene set, and used for normalization. The relative expression levels are depicted as the mean ± SD, which was calculated from three biological replicates.

The single (*ClUBCP* determined by NormFinder), pair (*ClEF1α* and *ClACT* identified by geNorm), and multiple best reference genes (determined by geNorm) identified in the abiotic stress subset were used for normalizing the expression of catalase genes under low temperature stress. geNorm analysis showed that five reference genes were required for more reliable normalization in the abiotic stress subset. The use of five reference genes to normalize three target genes is not feasible in practice. Therefore, Vandesompele et al. proposed that at least three best genes are required for more reliable normalization [11]. Accordingly, *ClEF1α*, *ClACT*, and *ClUBCP* were used to compose the multiple reference gene set for normalization. NF was calculated as the geometric mean of the used reference genes. The widely used reference gene *Cl18SrRNA* was ranked second from the bottom by both geNorm and NormFinder in the abiotic stress subset, and used as the control. The results are presented in Fig. 4.

All watermelon catalase family genes were induced by low temperature stress in leaves and showed different expression patterns. Transcripts of *ClCAT1* and *ClCAT2* were upregulated in the first 24 h, and subsequently declined. *ClCAT1* showed higher relative expression levels than *ClCAT2*. By contrast, the expression of *ClCAT3* was obviously downregulated when the best reference genes were used for normalization. The expression levels of catalase genes showed similar change patterns with slight differences when the identified single best reference gene of *ClUBCP*, pair of *ClEF1α* and *ClACT*, and multiple best reference gene set of *ClEF1α*, *ClACT*, and *ClUBCP* were used for normalization. Relative expression levels of the catalase genes that were normalized by the multiple reference gene set showed moderate changes compared with those of the single and pair of best reference genes. However, significantly different normalization results were observed between the best reference genes and unstable *Cl18SrRNA*. The relative expression levels of *ClCAT1* and *ClCAT2* were obviously overestimated when *Cl18SrRNA* was used for normalization. Moreover, an expression pattern of upregulation in the first 12 h and a subsequent downregulation was observed for *ClCAT3* when normalized by *Cl18SrRNA*, which completely differed from that for *ClCAT3* normalized by the best reference genes. The function of *ClCAT3* in response to low temperature stress was misinterpreted.

## Discussion

In previous gene expression studies on watermelon, only *18SrRNA* or *ACT* was used as a reference gene in qRT–PCR analyses. However, *18SrRNA* and *ACT* have not been systematically evaluated for their expression stability in watermelon, which is not in compliance with the MIQE guidelines [14], [23]. The accuracy of the qRT–PCR results is highly dependent on the robust normalization strategy of employing an invariant reference gene. No reference gene has been validated in watermelon for qRT–PCR analyses as of this writing, which has resulted in the misinterpretation of quantification results. Thus, validation of suitable reference genes for watermelon is required to ensure the accuracy of gene expression studies by qRT–PCR.

The selection of potential reference genes from the genes that had been validated in other crops is a good starting point and an efficient approach for identifying suitable reference genes for crops that lack validated reference genes [10]. The previously published reference genes in other crops, together with the frequently used reference gene *18SrRNA* in watermelon, were selected in this study to compare their expression stability under various experimental conditions to select the optimal reference genes for normalization in qRT–PCR analyses in watermelon.

Primer specificity is vital for reference gene validation [24]. Most of the commonly used reference genes belong to different gene families. Genes in the same family share conserved sequences. Designing specific primer pairs for a family of genes is difficult, particularly for crops without full genome sequences. When multiple family members are amplified, an increase in the expression abundance and variation in the tested gene may occur. To overcome this limitation in the present study, the generated primer pairs were aligned with all watermelon CDS to ensure their specificity on a genomic scale. Moreover, the possible gDNA contaminations in the samples can also introduce errors in the results. The strategy of at least one primer of a pair covering an exon-exon junction was adopted in *Arabidopsis* and tomato to overcome this problem [24], [25]. However, finding a suitable primer binding site in the limited region of exon-exon junction for many candidate genes is difficult. Therefore, the forward and reserve primer sequences were intentionally targeted on the neighboring exons separated by an intron to control possible genomic DNA contamination in the present study. This strategy was also successfully used in peanut [26]. All the designed primer pairs amplified a larger product on gDNA templates than on cDNA templates, which could be used to check for gDNA contamination in the cDNA samples. However, the most frequently used reference gene *18SrRNA* in watermelon was not powerful, and amplified the same product on cDNA and gDNA. The specific primer pairs developed in the study ensured the specificity and efficiency of qRT–PCR analysis for the selected reference genes, and offered a good starting point for other reference validation studies in watermelon.

An ideal reference gene should have stable expression in the developmental stages and under various experimental conditions. However, considering the obtained Cp values of the tested reference genes, an invariant expression level was not found among the 20 samples (Fig. 2), which highlights the importance of seeking appropriate reference genes via statistical approaches. The widely used tools geNorm and NormFinder were used to evaluate the expression stability of the candidate reference genes. Differences between the results of geNorm and NormFinder were found (Tables 2 and 3). The top seven stable genes (half of the total) were almost similar in the two algorithms for each sample set, but differences were found in the ranking order. Regardless of the changes in ranking order, the most unstable gene was the same for geNorm and NormFinder in each sample set.

In the present study, *ClYLS8* and *ClPP2A* were ranked as the best reference genes by geNorm in all 20 samples. However, when the samples were subdivided into different subsets based on experimental conditions, the best reference genes changed accordingly (Table 2). *ClTUA* and *ClACT*, *ClCAC* and *ClTUA*, *ClEF1α* and *ClACT* were identified as the best reference gene pairs by geNorm in the organs and tissues under normal growth conditions, biotic stress, and abiotic stress, respectively. A similar trend was also observed by NormFinder (Table 3). These changes illustrate the impossibility of compiling a list of suitable genes that can be used as references across a wide range of experimental conditions [6], which highlights the necessity of systematic validation of reference genes under every set of specific experimental conditions [27], [28]. However, data obtained by wide-scale gene expression analyses can be used as a starting point to choose candidates for the subsequent systematic validation of reference genes [10].

Reference gene validations have been performed in cucurbit crops, such as cucumber, melon, and zucchini. In cucumber, *EF1α*, *Fbox*, *CAC*, and *TIP41* are stable genes under different abiotic stresses, growth regulator treatments, and nitrogen nutrition treatments [29], [30]. *EF1α*, *UBIep*, and *TUA* are the suitable reference genes in another study on cucumber [31]. In zucchini, the combination of *UFP*, *EF1α*, *RPL36aA*, *PP2A*, and *CAC* genes is the best strategy for reliable normalization [32]. *RPL2* is stably expressed in melon stem infected with *Fusarium* wilt [33]. Similary, in the present study, the individual genes or combinations of *ClEF1α*, *ClTUA*, *ClCAC*, *ClUBCP*, *ClYLS8*, and *ClPP2A* were identified as the best reference genes by geNorm or NormFinder under different experimental conditions. However, considerable differences in the ranking of the candidate reference genes were also observed among the cucurbit crops. For example, *TUA* was found to be less stable in zucchini and cucumber [29], [32]. *EF1α* was found to be unsuitable in melon [33]. *YLS8* was the most variant gene in cucumber under different nitrogen nutrition treatments [30]. These discrepancies on the most and least stable genes highlight the species-specific and experimental condition-specific characteristics of reference genes [34]. To guarantee the accurate quantification of target gene expression, validating the expression stability of reference genes prior to their use for normalization in qRT–PCR data analysis should be performed.

Only a single reference gene was used for normalization in previous studies on watermelon gene expression. However, an increasing number of studies shows that using multiple reference genes for normalization can improve the reliability of results because a certain level of variation always exists for any reference gene [11], [35]. geNorm analysis in the present study indicated that two to nine reference genes were required for reliable normalization depending on the experimental conditions when the recommended cut-off value of 0.15 was used, which is infeasible particularly when a small number of target genes is tested. Vandesompele et al. recommended that the cut-off value of 0.15 should not be considered as a strict cut-off, and using three of the best reference genes is a valid normalization strategy in most cases [11]. Thus, at least three reference genes were needed for more reliable normalization of gene expression in watermelon.

*18SrRNA* has been widely used as a reference gene in different organs and tissues of watermelon under various experimental conditions. In this study, *18SrRNA* was ranked from second to seventh from the bottom in all samples and different subsets both by geNorm and NormFinder, which demonstrates that *18SrRNA* was not a suitable reference gene under specific experimental conditions and across all the tested experimental conditions. Moreover, *18SrRNA* exhibited a significantly higher expression level than other genes, which was unsuitable for normalization of target genes with middle or low expression levels. Therefore, the use of *18SrRNA* as a reference gene should be avoided in qRT–PCR analysis in watermelon in the future. *18SrRNA* is also unstable in cucurbit crops of melon [33], zucchini [32], and cucumber [31].

NormFinder usually generates a single best reference gene, whereas geNorm generates a pair of best reference genes and a set of multiple reference genes. To test the reliability of the identified reference genes, the expression levels of watermelon catalase family genes were quantified and normalized using the best reference genes, including the single, pair, and multiple best reference genes. The unstable and widely used *18SrRNA* in watermelon was also used for normalization. The expression of watermelon catalase family genes showed similar change patterns when the best reference genes were used for normalization. Compared with the single and pair of best reference genes, moderate changes in the expression levels were observed for the catalase genes when the multiple reference gene set was used for normalization, which indicates that multiple reference genes resulted in more reliable normalization results than single reference genes. However, significantly higher expression levels and different expression patterns were observed for these genes when *18SrRNA* was used for normalization, which resulted in overestimation and misinterpretation of the transcripts of catalase family genes. Similar results were also observed in other crops [36]–[39]. Thus, the selection of reference genes greatly affected the normalization results, and an inappropriate reference gene may introduce bias in the analysis and lead to misleading results.

Catalase genes encode a small family of proteins, which can catalyze the decomposition of H_2_O_2_ and have important functions in controlling ROS homeostasis. Watermelon originated in Africa and is susceptible to low temperature stress. Analysis of the expression profiles of catalase genes in leaves under low temperature stress is crucial to reveal their regulatory functions. Differential expression patterns in response to low temperature were observed among catalase genes in watermelon. Upregulations of *ClCAT1* and *ClCAT2* in the first 24 h indicate that they had major functions in the removal of H_2_O_2_ generated under low temperature stress, whereas transcriptional downregulation of *ClCAT3* could sustain increased H_2_O_2_ availability, which was necessary for ROS homeostasis. Similar results were also observed in *Arabidopsis* [18].

Thus, the identified reference genes in the present study outperformed the currently used reference gene *18SrRNA* in watermelon in terms of expression stability during plant development and under different environmental conditions. Suitable reference genes should be selected depending on the experimental conditions. Multiple reference genes are recommended for more reliable normalization of gene expression in watermelon. In addition, the identified reference genes were expressed at much lower levels than *18SrRNA*, making them highly suitable for normalization of gene expression over a wide range of transcript levels. The identified reference genes with their specific primers will lead to better normalization and quantification of transcript levels in watermelon in the future.

## Supporting Information

Figure S1 **Melting curve analyses on the candidate reference genes.**
(PDF)

Figure S2 **Amplification specificity of watermelon genes belonging to the catalase family, as confirmed by electrophoresis in 2% agarose gel and melting curve analysis.**
(PDF)

Table S1 **Description of watermelon catalase family genes.**
(PDF)
