# Supplementary material for: Identification of Suitable Reference Genes for Gene Expression Normalization in qRT-PCR Analysis in Watermelon
Source: PLoS One. 2014 Feb 28;9(2):e90612. doi: 10.1371/journal.pone.0090612 (PMC3938773; doi:10.1371/journal.pone.0090612)
Supplement: Figure S1 — Melting curve analyses on the candidate reference genes. (PDF) [file pone.0090612.s001.pdf]

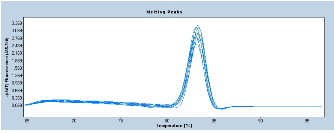

ClACT

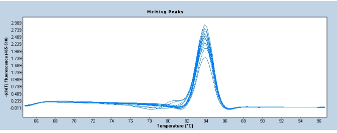

ClCAC

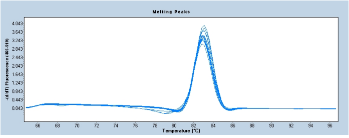

ClEF1α

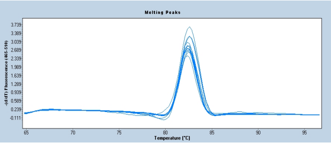

ClGAPDH

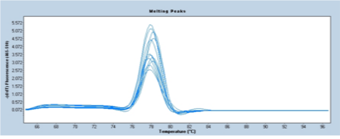

ClIDH

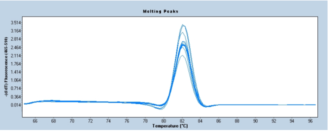

ClLUG

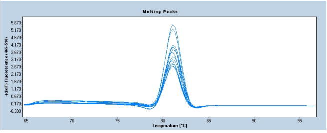

ClPP2A

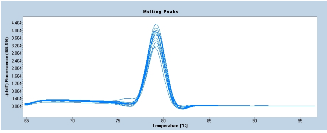

ClPTB

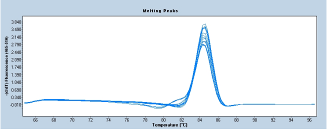

ClRPS2

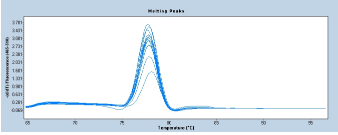

ClSAND

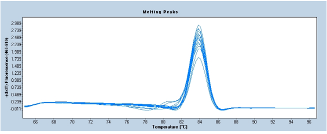

ClTUA

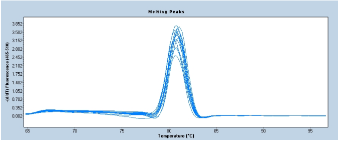

ClUBC2

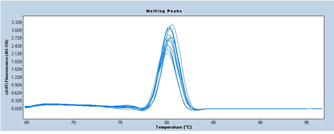

ClUBCP

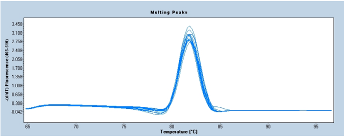

ClYLS8

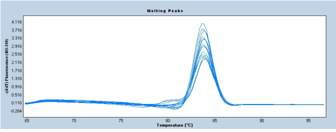

Cl18SrRNA

Fig. S1 Melting curve analyses on the candidate reference genes
