# Supplementary material for: Identification of Suitable Reference Genes for Gene Expression Normalization in qRT-PCR Analysis in Watermelon
Source: PLoS One. 2014 Feb 28;9(2):e90612. doi: 10.1371/journal.pone.0090612 (PMC3938773; doi:10.1371/journal.pone.0090612)
Supplement: Figure S2 — Amplification specificity of watermelon genes belonging to the catalase family, as confirmed by electrophoresis in 2% agarose gel and melting curve analysis. (PDF) [file pone.0090612.s002.pdf]

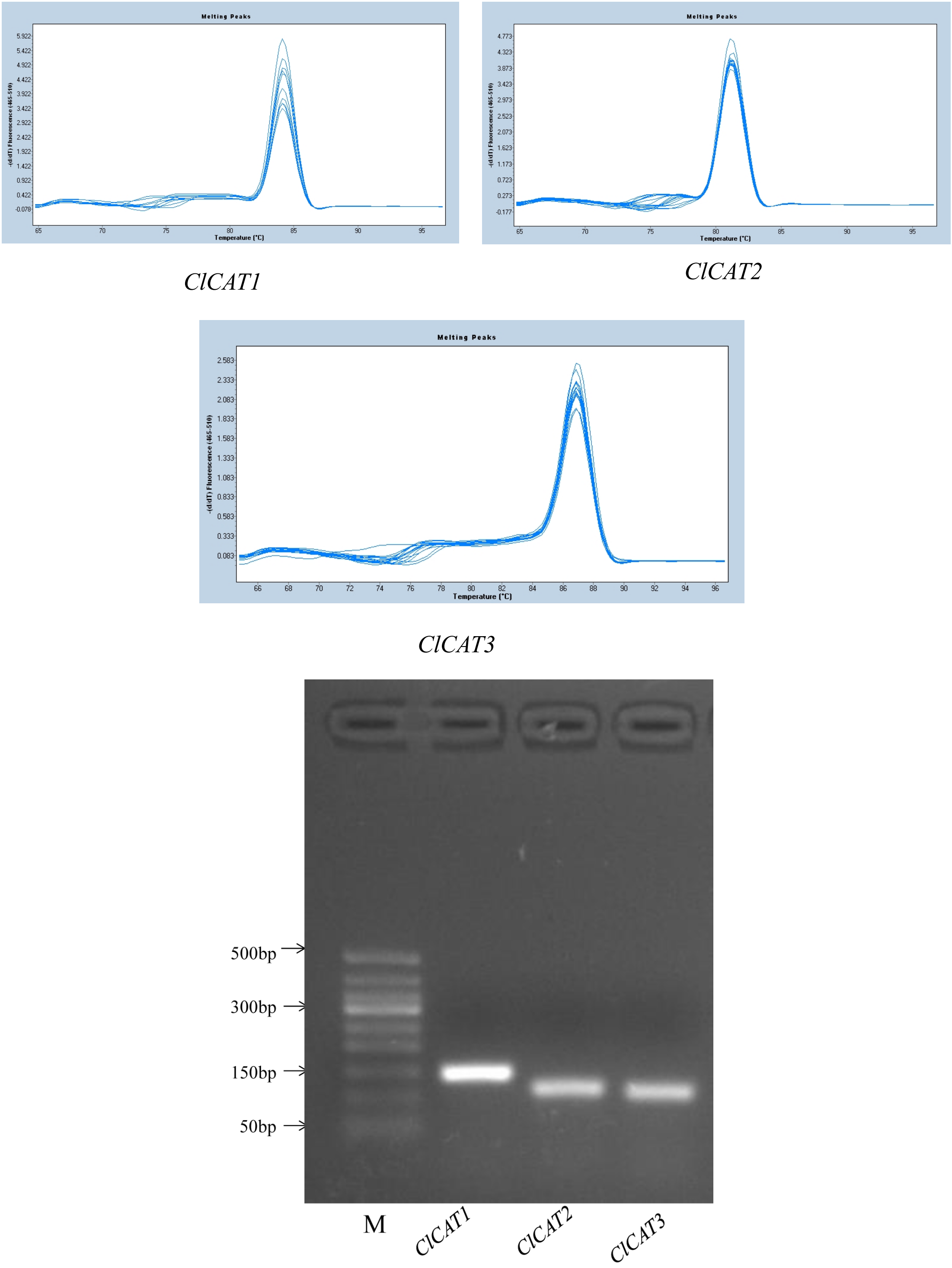

Fig. S2 Amplification specificity of watermelon genes belonging to the catalase family, as confirmed by electrophoresis in 2% agarose gel and melting curve analysis
