# Supplementary material for: Identification of Suitable Reference Genes for Gene Expression Normalization in qRT-PCR Analysis in Watermelon
Source: PLoS One. 2014 Feb 28;9(2):e90612. doi: 10.1371/journal.pone.0090612 (PMC3938773; doi:10.1371/journal.pone.0090612)
Supplement: Table S1 — Description of watermelon catalase family genes. (PDF) [file pone.0090612.s003.pdf]

**Table S1** Description of watermelon catalase family genes

| Gene name | Gene ID[a] | Arabidopsis homolog locus[b] | E-value | Identitiy[c] | Forward primer seqence 5'-3' | Reverse primer seqence 5'-3' | Product size (bp) |
|---|---|---|---|---|---|---|---|
| *ClCAT1* | Cla023448 | AT1G20630 | 0 | 87% | F:GCTCACCATGCCGAGAGGTATC | R:CGTTCTTGCCTGTCTGATGTCC | 137 |
| *ClCAT2* | Cla023447 | AT4G35090 | 0 | 92% | F:ACAACTCCTCGATGACCGTTGG | R:GACAACACGCTCTGGAATACGC | 110 |
| *ClCAT3* | Cla021932 | AT1G20620 | 0 | 71% | F:TTCTCCACCGTCATCCACGAG | R:CTAAGTCGAAGTTGCCTTCACG | 100 |

a, Watermelon gene ID in Cucurbit Genomics Database (http://www.icugi.org)

b, Arabidopsis gene ID in TAIR database (http://www.arabidopsis.org/)

c, The identity data are the results of blastp.
